# Influence of the Carbohydrate Moieties on the Immunoreactivity and Digestibility of the Egg Allergen Ovomucoid

**DOI:** 10.1371/journal.pone.0080810

**Published:** 2013-11-14

**Authors:** Sara Benedé, Rosina López-Fandiño, Marta Reche, Elena Molina, Iván López-Expósito

**Affiliations:** 1 Departamento de Bioactividad y Análisis de Alimentos, Instituto de Investigación en Ciencias de la Alimentación, Consejo Superior de Investigaciones Científicas, Universidad Autónoma de Madrid, Madrid, Spain; 2 Servicio de Alergia, Hospital Infanta Sofía, San Sebastián de los Reyes, Madrid, Spain; University of Insubria, Italy

## Abstract

**Background:**

Ovomucoid (OM) has two carbohydrate chains on each of the first and second domains and one in the third. The contribution of the covalently bound carbohydrate chains to the overall OM allergenicity is controversial. Another aspect directly related with the immunological properties of OM that has not been studied in depth is the importance of the carbohydrate chains on its digestibility.

**Objective:**

The aim of the study was to assess the involvement of the carbohydrate moieties of OM in its digestibility and allergenic properties.

**Methods:**

IgE-binding and basophil activation by glycosylated and enzymatically deglycosylated OM (dOM) were compared using blood from egg-allergic patients. The peptides obtained after digestion using a physiologically relevant model were identified by RP-HPLC-MS/MS and the IgE-binding of the resulting fragments was evaluated by DOT-Blot.

**Results:**

No structural changes were observed after deglycosylation of OM. 80% of the patients showed lower IgE binding to dOM as compared with OM and, in some patients, IgE reactivity could not be inhibited by pre-incubation with dOM. A subtle reduction in the percentage of activated basophils was observed when incubated with dOM as compared to OM. Following simulated digestion, dOM was more extensively degraded than OM, particularly during the gastric phase and both, OM and dOM, yielded, after the duodenal phase, immunoreactive fragments that were totally or partially coincident with previously described epitopes.

**Conclusion:**

*&* *Clinical* *Relevance*: this work demonstrated an enhanced IgE reactivity towards carbohydrate containing OM in some egg-allergic patients that could be attributed to cross-sensitization or sensitization to the glycosylated components. The carbohydrate chains contributed to an increased resistance to proteolysis, and thus, to its allergenic potency. Evaluation of the products of digestion of OM and dOM revealed the presence of high-frequency IgE-binding epitopes that could remain linked by disulphide bonds.

## Introduction

Egg allergy is the second most common food allergy with a prevalence of up to 1.7 % of children and adults on the basis of the available studies involving double-blind placebo-controlled food challenges [1]. It is typically identified in infancy, with patients susceptible of developing every disease of the allergic march, including eczema, aeroallergen sensitization and asthma [2]. Clinically, symptoms may vary in severity from atopic dermatitis to systemic anaphylaxis, representing the latter an important risk when administering certain vaccines that contain egg derivatives as excipients [3]. 

Egg white is the main source of allergens in egg. Four of them, named from Gal d 1 to Gal d 4, have been identified as the major ones, with ovomucoid (OM) (Gal d 1), which makes up to 11% of the egg white, being considered the immunodominant as judged by its binding frequency to IgE from allergic patients. OM from hen egg is a glycoprotein with trypsin inhibitor activity, a molecular mass of approximately 28.0 kDa, and an isoelectric point of 4.1. Its polypeptide chain consists of 186 amino acids, forming three structurally independent tandem domains each of 60 amino acids in length [4]. Each domain bears multiple conformational and linear epitopes that are recognized by IgE antibodies from egg allergic patients [5]. 

One particular characteristic of OM is its high carbohydrate content, which is between 20–25%, with two carbohydrate chains on each of the first and second domains and one chain present on about 50% of the third domain [4]. Thus, a relevant question that arises is whether the covalently-bound carbohydrate moieties contribute to OM allergenicity. Using sera from allergic patients, Matsuda et al. [6] reported that, in the third domain, the carbohydrate chain and/or its attachment site could be recognized as antigenic determinants, whereas Zhang and Mine [7] postulated that the carbohydrate moiety of the third domain rather exerted an inhibiting effect on the IgG and IgE binding properties of OM. Besler et al. [8] and Cooke and Sampson [5] concluded that the carbohydrate residues did not take part in the allergenic structures of OM. Hence, the issue of the relevance of the carbohydrate moiety of OM on its potential to sensitize or elicit an allergic response is still under debate. 

In the case of many food allergies, particularly to plant proteins, antibodies specific to carbohydrate determinants are frequently found, although they appear not to have clinical relevance [9]. In any case, the fact that glycosylation is a common feature to many food allergens has prompted investigations that showed that glycans may enhance immunogenicity through the activation of innate Th2 responses [10]. Furthermore, the carbohydrate chains normally exert a stabilizing effect on protein structure, offering protection towards processing and/or gastroduodenal digestion and thus contributing to the allergenic potential [11]. Regarding OM, there are a few studies dealing with the influence of gastrointestinal digestion on its immunoreactivity [12-14], however, the contribution of the glycan moieties to its digestibility has not been addressed.

The aim of the present work was to assess the involvement of the carbohydrate moieties of OM in its allergenic properties and digestibility. To that end, OM was enzymatically deglycosilated and, using blood from egg-allergic patients, the IgE-binding and basophil activation properties of the glycosylated and deglycosylated forms were compared. Glycosylated and deglycosylated OM were hydrolysed with a physiologically relevant model by mimicking three areas of the gastrointestinal tract: the mouth, stomach and small intestine, the nonglycosylated peptides obtained were identified by RP-HPLC-MS/MS and the IgE-binding properties of the most relevant resulting fragments were evaluated.

## Materials and Methods

### Ethics statement

All human samples were obtained with written consent from the donors (in case of adults) or from the next of kin, caretakers, or guardians on the behalf of the minors/children involved in the study. The Bioethics Committee from the Consejo Superior de Investigaciones Científicas (CSIC) approved all experiments.

### Ovomucoid deglycosylation

OM from chicken egg white (Sigma-Aldrich, St. Louise, MO) was dissolved in 5 mM potassium phosphate, 4 mM CaCl_2_, 0.04% NaCl, pH 7.5, at a concentration of 20.7 mg/mL and deglycosylated with PNGase F (500 U/mL, Sigma-Aldrich, 1 U/0.8 mg of OM) at 37 °C, with constant stirring for 24h. PNGase F was added again to the solution at the same ratio and incubated for a further 4 days at 37 °C. A control without PNGase F was also included.

Free sugars were removed from deglycosylated OM (dOM) by centrifugation at 4000g for 5 min at 4 °C in ultrafiltration devices of 10.000 Da cut off (Millipore, Bedford, MA), until no absorbance was detected at 490 nm in the permeates [15]. Protein concentration was measured by the Pierce® BCA Protein Assay Kit (Pierce Scientific, Rockford, USA).

### SDS-PAGE Analysis

SDS-PAGE of OM, dOM and their digests was performed on Precast Criterion 4-12% and 12% Bis-Tris gels (Bio-Rad, Richmond, CA, USA) and electrophoretic separations were carried out at 150 V, using XT-MES as running buffer (Bio-Rad), Samples were diluted to 1 and 5 mg/mL in sample buffer containing 2% (w/v) SDS and 5% (v/v) β-mercaptoethanol and heated at 95°C for 4 min. Gels were stained with Bio-Safe Coomassie G-250 (Bio-Rad) or Periodic Acid Schiff (PAS).

### Circular Dichroism

Circular dichroism (CD) spectra were obtained in a Jasco J-810 spectropolarimeter (Jasco Corp., Tokyo, Japan). Far (195-260 nm) and near (250-350 nm) UV CD spectra of OM and dOM, in phosphate buffer 50 mM pH 7.0, were recorded at 20 °C using cells with respective path lengths of 0.1 and 0.2 cm. Spectra represent the average of three accumulations collected at 20 nm/min, with a 2 s time constant, a 0.2 nm resolution, and a sensitivity of 100 mdeg. The samples were dissolved at 0.2 mg/mL for the analysis in the far-UV region and at 0.5 mg/mL for the near-UV region. The buffer blanks were subtracted from each CD spectrum. Empirical determinations of protein secondary structure were obtained employing the CDNN secondary structure analysis software (Applied Photophysics Ltd, Leatherhead, Surrey, UK). 

### Human IgE Binding by Inhibition ELISA

Human IgE-binding of OM, dOM and their digests was assessed by inhibition ELISA (using OM as coating antigen) as previously reported [16]. Individual serum samples from children with proven allergy to egg proteins and specific IgE antibodies towards OM (OM-IgE), as determined by the FEIA-CAP System (Pharmacia Diagnostics, Uppsala, Sweden) (Patients 1-16, Table 1). 

**Table 1:** Specific IgE levels (kU/L) towards egg white, yolk and ovomucoid of the sera used in the inhibition ELISA, Western blotting, dot blot, and basophil activation experiments.

**Patient**	**Age (years)**	**IgE levels (kU/L)**		
		**White**	**Yolk**	**OM**
1	11	54.7	32.9	37.1
2	7	27.6	20.6	41.1
3	7	41.7	25.7	61.5
4	3	73.2	42.5	62.2
5	12	66	42.9	70.4
6	9	92.7	63.8	77.4
7	7	>100	>100	90.7
8	3	>100	90.9	>100
9	6	>100	>100	>100
10	8	>100	>100	229
11	3	75.9	14.1	46.6
12	7	36.7	5.1	48.3
13	6	>100	>100	87.8
14	6	11.6	4.32	12
15	6	40.4	15.8	49.5
16	4	50.9	42.1	62.1

### Western Blotting

Western Blotting following SDS-PAGE of OM and dOM and their hydrolysates was performed as described [16], using the individual serum samples from patients 1-10 (Table 1). For Western-blot inhibition experiments, serum samples were pre-incubated with dOM for 2 h. The amount of dOM was calculated from inhibition ELISA assays (see above), as the concentration which inhibited 100% of IgE binding.

### Basophil activation test

Basophil activation assays were performed as described by Martos et al [17], with some modifications. Peripheral blood mononuclear cells (PBMCs) from 6 egg-tolerant adult donors were isolated according to standard procedures [18]. Then, they were stripped from bound IgE by treatment with lactic acid and re-sensitized with a 1:1.5 dilution of a pool of sera from 5 children with egg allergy (patients 1, 3, 5, 7 and 9, Table 1). Subsequently, they were incubated with OM and dOM at a range of concentrations from 0.01 to 10 μg/mL. Cells were stained for CD63, CD123 (BD Biosciences, Franklin Lakes, NJ, USA), HLA-DR, and CD203c (Beckman Coulter, Indianapolis, IN, USA), fixed with BD FACS^TM^ Lysing Solution (BD Biosciences), and acquired in a Gallios Flow Cytometer (Beckman Coulter). Anti IgE antibody (Dako Denmark., Glostrup, Denmark) and fMLP (Sigma-Aldrich) were used as positive control. Percentage of activation upon stimulation with OM and dOM was calculated and normalized according to the value obtained for RPMI-IL3 (negative control). 

### In vitro orogastroduodenal digestion

For *in vitro* oral digestion, OM and dOM were dissolved in simulated saliva fluid (5 mM potassium phosphate, 4 mM CaCl_2,_ 0.04% NaCl, pH 6.5) at a concentration of 18.8 mg/mL. After incubation at 37 °C for 15 min, α-amilase (EC 3.2.1.1, 210 U/mg solid, Sigma-Aldrich) was added at the physiological ratio of 150 U/mL of simulated fluid. Oral digestions were performed at 37 °C during 2 min and stopped by decreasing the pH to 3.5 with 1N HCl.

*In vitro* gastric and duodenal digestions were performed according to [16] with some modifications. For gastric digestions, simulated gastric fluid (35 mM NaCl, pH 2.0) containing phospholipid (9.58 mg/mL) vesicles was prepared according to [19]. Gastric digestions were conducted at pH 2.0, for 60 min at 37 °C with 182 U/mg OM of porcine pepsin (EC 3.4.23.1, 3640 U/mg protein, Sigma-Aldrich), using the two-min oral digests as the starting material. Aliquots were withdrawn at different time points up to 60 min and the digestions were stopped by increasing the pH to 7.5 with 1M NaHCO_3_.

 Duodenal digestions were performed on the 60 min gastric digests re-adjusted to pH 6.5, with the addition of 0.25 M Bis-tris, pH 6.5, 1 M CaCl_2_ and 0.250 M bile salt mixture. After preheating at 37 °C for 15 min, pancreatic porcine lipase (EC 232-619-9; type VI-s, 47900 U/mg protein), pancreatic porcine colipase (EC 259-490-1), pancreatic bovine trypsin (EC 232-650-8; type I, 10100 BAEE U/mg protein), and pancreatic bovine α-chymotrypsin (EC 232-671-2; type I-s, 55 U/mg protein) (all from Sigma-Aldrich) were added to the duodenal mix [20]. The reactions were carried out at 37 °C for different time points up to 30 min and stopped by adding a solution of Bowman–Birk trypsin-chymotrypsin inhibitor (Sigma Aldrich). The final composition of the mixture was 3.3 mg/mL phosphatidylcholine, 3.9 mg/mL OM, 7.4 mM bile salts, 7.6 mM CaCl_2_ and 20.3 mM Bis-tris.

### Peptide sequencing by RP-HPLC-MS/MS

RP-HPLC-MS/MS analyses of the digested samples, after a reducing step using 70 mM dithiothreitol (DTT) at pH 7.0 for 1 h at 37 °C, were performed on an Agilent 1100 HPLC System (Agilent Technologies, Waldbron, Germany) with a RP318 C18 column (250 x 4.6 mm, Bio-Rad, Richmond, CA, USA). The HPLC system was connected on-line to an Esquire 3000 quadrupole ion trap (Bruker Daltonik, Bremen, Germany) equipped with an electrospray ionisation source. Operating conditions were as follows: solvent A, 0.37 mL/L TFA in Milli-Q water and solvent B, 0.27 mL/L TFA in HPLC grade acetonitrile; flow rate, 0.8 mL/min; injection volume, 50 μL. A linear gradient of solvent B in A, from 0 to 60% in 60 min, followed by 60% B for 30 min was used. Mass spectra were recorded over the range 100–3000 m/z using Data Analysis^TM^ (version 4.0, Bruker Daltoniks, Bremen, Germany). The m/z spectral data were processed and transformed to spectra representing mass values. BioTools (version 3.1, Bruker Daltoniks, Bremen, Germany) was used to process the MS(n) spectra and perform peptide sequencing.

### Dot-Blot

17 peptides selected from in vitro digestions were commercially synthesized (JPT peptide Technologies, Berlin, Germany). Among the deglycosylated peptides identified in the gastroduodenal digests of OM and dOM, the peptides shorter than 8 amino acids, those which were part of longer sequences and/or the peptides that overlapped with well-established epitopes of OM by microarray and SPOT membrane in previous studies, were discarded. Peptides with 8 and 9 amino acids were extended to 10 amino acids with the preceding and following amino acids of the protein chain and the peptides over 20 amino acids were replaced by 2 shorter overlapping peptides. 

The nitrocelullose membranes were conditioned in Tris buffer (48 mM Tris, 39 mM glycine, 20% methanol, pH 9.2) for 20 min, and 1µl from each peptide was spotted onto the membrane and allowed to dry. Then, the nitrocellulose membranes were blocked with Tris Buffer Saline containing 0.05% v/v Tween 20 (TBST, pH 7.6) with 1% w/v BSA, for 60 min and washed with TBST. Immunolabelling with ten individual serum samples (patients 1-10, Table 1) was conducted as described for the Western Blotting experiments.

### Statistical analysis

All data was analyzed with Prism (Version 5; GraphPad, La Jolla, CA, USA). Student t-test was used to compare between groups. Data were considered statistically significant for P values less than 0.05.

## Results

### Deglycosilation of OM

**Figure 1:**
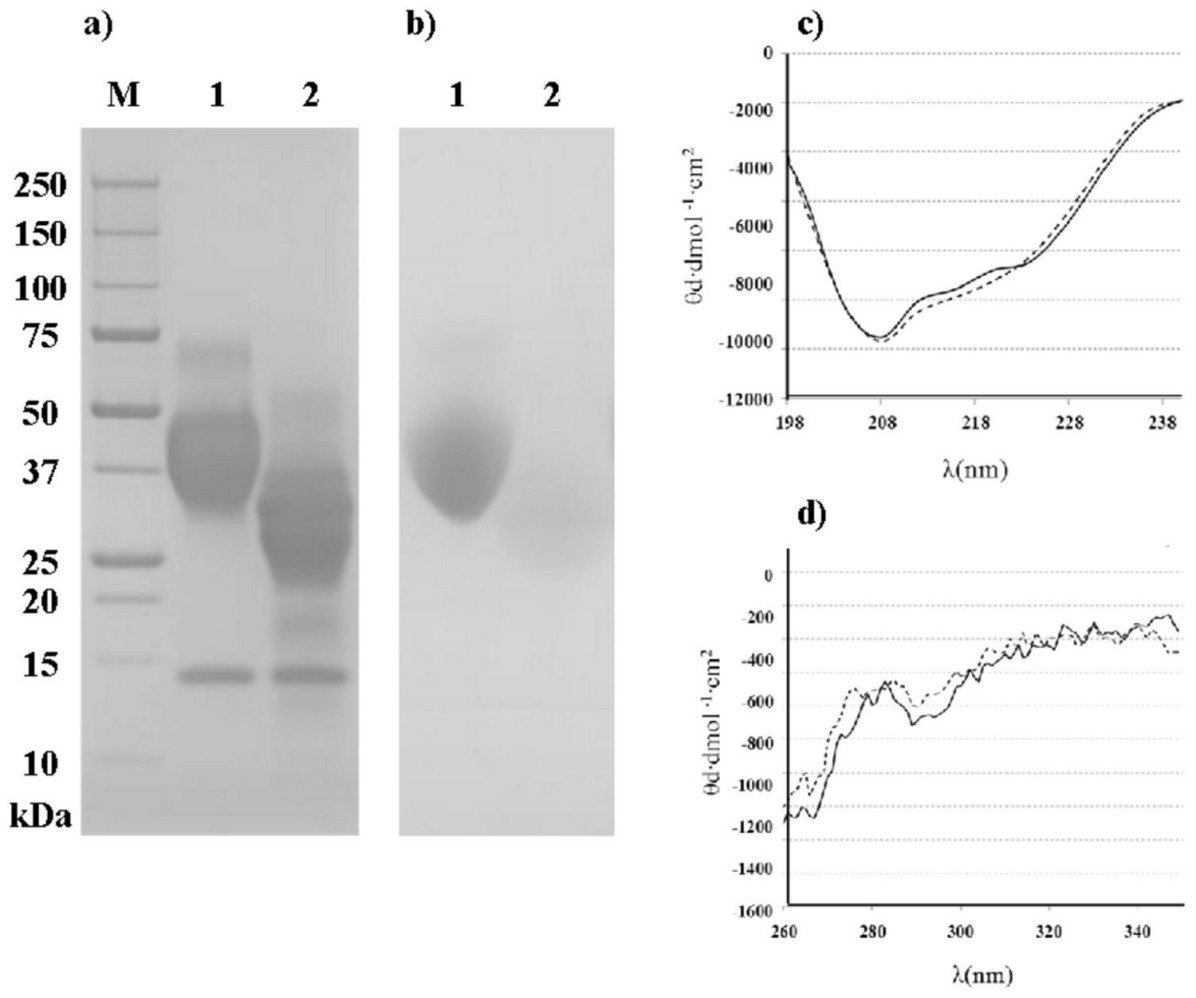
OM deglycosilation results. SDS-PAGE patterns of OM and its deglycosylated form (dOM) with Coomassie blue (a) or PAS (b) staining M: molecular mass marker; 1: OM, 2: dOM. Circular dichroism spectra in the far (c) and near (d) UV region of OM (−) and dOM (---). The spectra were recorded in phosphate buffer, pH 7.0, at 20°C.

To assess the effectiveness of deglycosylation SDS-PAGE with Coomassie G-250 and PAS staining was conducted. As shown in Figure 1a, OM appeared as a diffuse group of bands from 30 to 50 kDa, together with a protein band of 14 kDa corresponding to hen egg lysozyme, present due to an incomplete purification of the commercial product [21]. The electrophoretic pattern of OM was modified after treatment with PNGase F. The bands corresponding to dOM showed lower molecular mass and, consequently, higher electrophoretic mobility due to the removal of the carbohydrate chains. The absence of carbohydrate was confirmed by staining the gel with PAS (Figure 1b), which showed no bands in the lane corresponding to dOM.

 We further checked by CD-spectroscopy that, in addition to an efficient removal of N-linked oligosaccharides, PNGase F treatment maintained the degycosylated protein in its native structure. The far-UV CD spectrum of OM was similar to that reported by [22] and did not change by deglycosylation (Figure 1c). The estimated secondary structure percentages obtained were identical for α-helix (16%) and random coil (36%) features, and slightly different for β-sheet and β-turn (24 and 25% and 23 and 22% for OM and dOM, respectively). The near-UV CD spectra of native OM was not modified either (Figure 1d), revealing that the secondary and tertiary structures of dOM were not altered when the carbohydrate chains were cleaved.

### In vitro immunoreactivity

**Figure 2:**
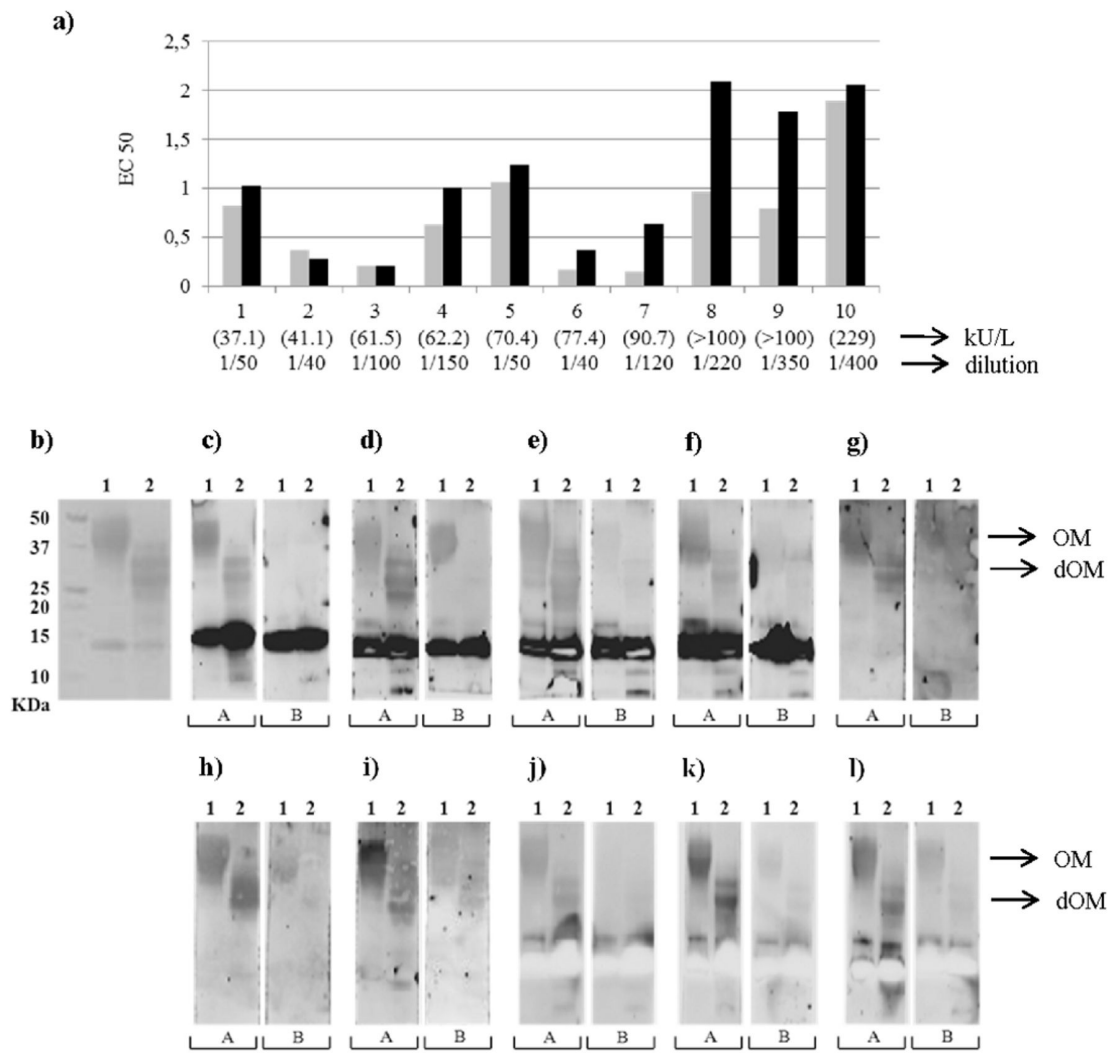
OM and dOM immunoreactivity . Binding to human IgE, using sera from 10 egg-allergic individuals (patients 1-10, Table 1), of OM (gray) and dOM (black), expressed as EC50, or effective OM or dOM concentration (μg/mL) to inhibit 50% of the IgE binding to OM (a). SDS-PAGE (b) and Western blot analyses (c-l) of OM (1) and dOM (2) with sera from the same 10 individuals (1-10, respectively, used in the ELISA). Human sera were either no incubated (A) or previously incubated (B) with the amount of dOM calculated to inhibit 100 % of IgE binding.

IgE-binding to OM and dOM was evaluated by inhibition ELISA using the sera from 10 egg-allergic patients (1-10 in Table 1). As depicted in Figure 2a, in 8 out of 10 sera the immunoreactivity towards dOM was lower than that of OM. 

In order to investigate the IgE specificity, sera from the same 10 patients were used to perform Western blot analyses (Figure 2c-l). IgE from the 10 sera recognized the bands corresponding to both OM and dOM. However, a previous incubation with dOM completely inhibited the binding to dOM but failed to inhibit the binding to OM in two patients (2 and 6, Figure 2d and 2h) and inhibited the binding to dOM to a much higher extent than that of OM in another two (9 and 10, Figure 2k and 2l). This provided evidence for the presence of IgE reactive epitopes specific for the glycosylated protein that did not react with the deglycosylated form. In the remaining 6 patients, preincubation with dOM either completely inhibited the IgE reactivity against dOM and OM (patients 1 and 8, Figure 2c and 2j), or partially inhibited both to a similar extent (patients 3, 4, 5 and 7; Figures 2e, 2f, 2g and 2i). The strong reactivity of many of the individual sera towards the contaminating lysozyme was noteworthy. 

### Ex vivo biological activity

**Figure 3:**
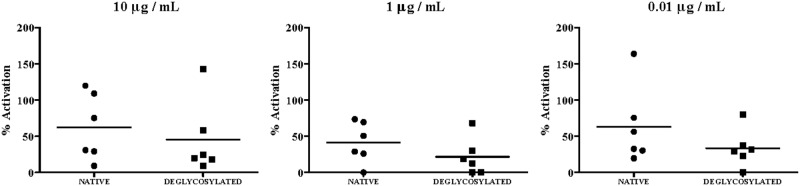
Basophil activation assays. Percentage of activated basophils (CD63^+^CD123^+^CD203c^+^HLA-DR^low^) after stimulation with OM and dOM at concentrations between 10 to 0.01 μg/mL. PBMCs from 6 egg-tolerant adult donors were passively sensitized with a pool of sera from 5 children with egg allergy (patients 1, 3, 5, 7 and 9, Table 1). Bars indicate the mean of activated basophils.

The capacity to trigger basophil activation was determined on PBMCs from 6 egg-tolerant adult donors passively sensitized with a pool of sera from patients 1, 3, 5, 7 and 9 (Table 1). The results of the basophil activation analysis by flow cytometry are shown in Figure 3. Challenge of basophils with OM and dOM induced activation as measured by upregulation of CD63. Results showed that the removal of the carbohydrate chains moderately reduced the percentage of activated basophils at all the concentrations assayed, although the differences did not reach statistical significance.

### In vitro simulated orogastrointestinal digestion and immunoreactivity

**Figure 4:**
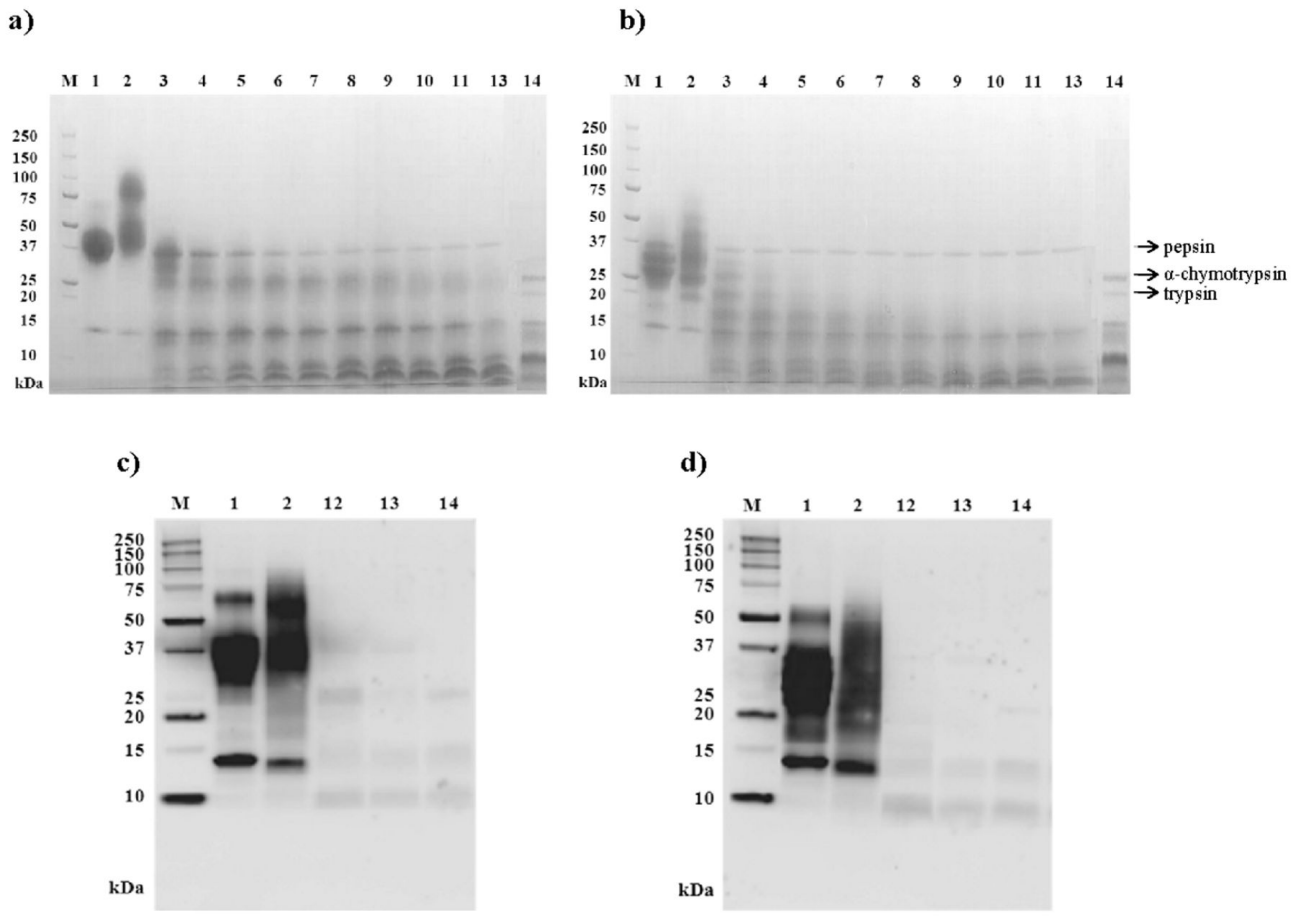
Orogastrointestinal digestions profile and immunoreactivity. SDS-PAGE patterns (a, b) and Western blotting (c, d) of OM (a, c) and dOM (b, d) after in vitro oral, gastric and gastroduodenal digestions. M: molecular mass marker; 1: OM (a, c) and dOM (b, d); 2: oral digest; 3-13: gastric digests at 1, 2, 3, 4, 5, 7, 10, 15, 20, 30 and 60-min; 14: duodenal digests (60 min of gastric digestion followed by 30 min of duodenal digestion). Membranes were incubated with a pool of sera (patients 1, 3, 5, 7 and 9, Table 1).

Figure 4a and 4b show the SDS-PAGE patterns of OM and dOM following the *in vitro* oral, gastric and duodenal digestions. After the 2 min-oral phase, the SDS-PAGE patterns from both OM and dOM showed a much broader shape, which could be attributed to an incomplete reduction of the nine disulfide bridges caused by the low sample pH (pH 3.5 was used to stop α-amilase action), which diminished their mobility in the acrylamide gel [23]. However, as judged by the RP-HPLC pattern (not shown), there were no changes after 2 min of hydrolysis that would point to an unspecific activity of the saliva enzyme. OM was degraded during the first minutes of gastric digestion, leaving no intact protein at the end of the gastric phase, and yielding fragments with molecular masses of ~25, ~15 and <10 kDa. All these bands were faintly visible after 60 min of simulated gastric hydrolysis and the lower molecular mass ones (~15 and <10 kDa) persisted throughout the duodenal phase (not shown). dOM was degraded more rapidly and produced bands of ~18, ~ 15 and <10 kDa, with the band corresponding to ~18 kDa being no longer present after the first 7 min of pepsin hydrolysis, so that only the bands corresponding to ~15 and <10 kDa were found at the end of gastric digestion and throughout the subsequent duodenal digestion. 

As shown in Figure 4c and 4d, the ~25, ~15 and <10 kDa fragments formed during gastric digestion of OM and those of ~15 and <10 kDa formed during gastric digestion of dOM were able to bind IgE from egg-allergic patients, although the immunoreactivity of the band of ~15 kDa could be, at least partially, attributed to the presence of residual LYS. Once the duodenal digestion was completed, the bands corresponding to ~15 and <10 kDa, present in OM and dOM digests, still had detectable IgE-binding capacities. 

The IgE-binding of the digests was evaluated by inhibition ELISA using the sera from patients 11-16 (Table 1). The immunoreactivity decreased to, approximately, 10.6% for OM and 1.2% for dOM at the end of the gastric stage, falling to 4.16% and 1.1% after the duodenal phase for OM and dOM, respectively. The lower IgE binding found in the digests of dOM correlated with its highest susceptibility to proteolysis.

### Peptide identification *after* in vitro digestion and epitope recognition

**Figure 5:**
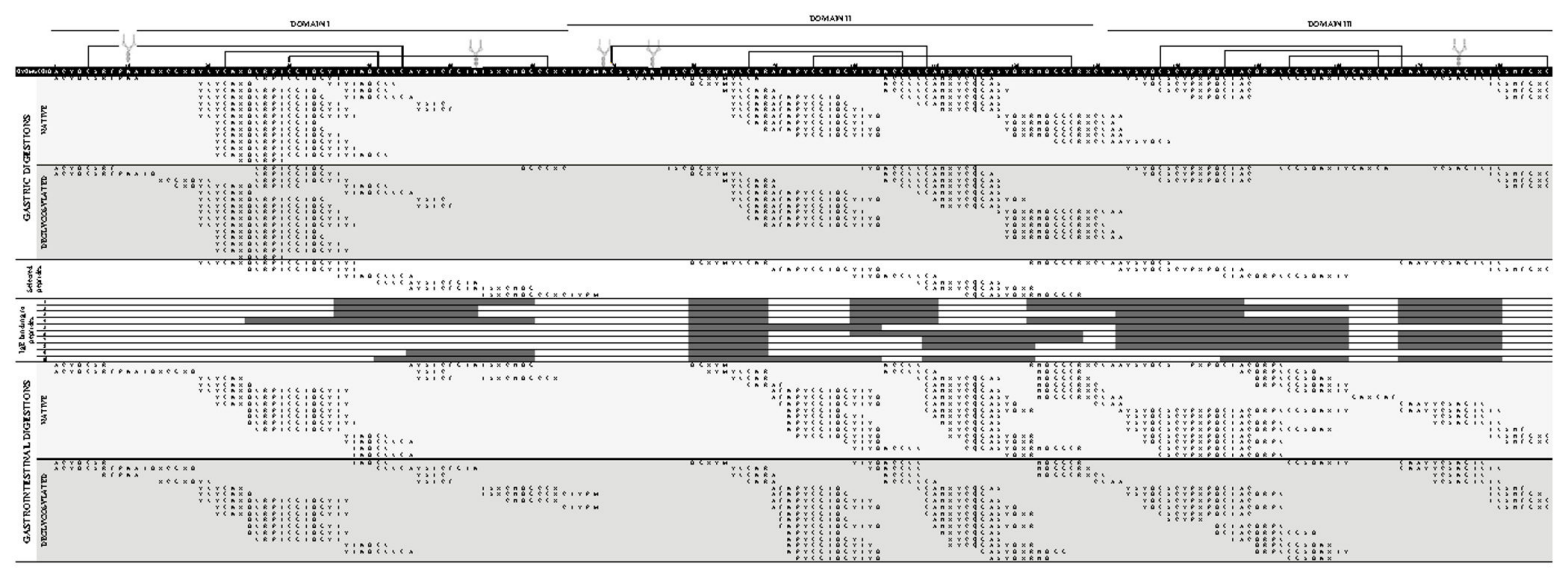
Peptide identification and IgE binding properties. Peptide sequences, identified by RP-HPLC-MS/MS, in the gastric (60 min) and subsequent duodenal digests of OM and dOM (60 min of gastric followed by 30 min of duodenal digestion), and IgE binding, estimated by immunodot with 10 sera of egg-allergic patients (patients 1-10, Table 1), of 17 synthetic peptides selected within the peptides identified in the simulated digestions.

RP-HPLC-MS/MS was employed to analyze the peptides present in the gastric (60 min) and duodenal (30 min) digests of OM and dOM and Figure 5 shows the sequences of the 146 peptides identified. Only identification of the non-glycosylated fragments was attempted, in view of the difficulty involved in the determination of glycosylated peptides in complex mixtures, because the signal intensity of glycopeptides is low compared to nonglycosylated peptides and often suppressed in their presence [24]. Some deglycosylated peptides containing Asn_10_ and Asn_75_ were detected in the OM hydrolysates (Asn_175_ is naturally partially glycosylated) probably due to a partial deglycosylation of the commercial sample used. The peptide patterns of the hydrolysates produced were very similar, showing 56 peptides in common after the simulated orogastrointestinal digestion. This indicated that, while deglycosylation enhanced susceptibility to proteolysis, no major differences in the cleavage sites of the protein were detected after hydrolysis times representative of the transit times in the stomach and duodenum. In the N-terminal region, around the glycosylated Asn_10_, pepsin hydrolysed OM after Ala_12_ and dOM after Phe_8_, Asp_13_ and Glu_15_. Very few peptides were identified in the region between the glycosilated positions Asn_53_ and Asn_75_ even in the deglycosylated form (Figure 5). In this area, pepsin hydrolysed dOM after His_58_ and Asp_64_ and trypsin after Lys_63_, in both OM and dOM. Met_68_ was also cleaved in the duodenal digests of dOM. The greatest similarities were found in the third domain, particularly following gastric digestion. Interestingly, peptides with a molecular weight above 2400 Da only appeared during the digestion of OM, underlining a less extensive proteolytic degradation.

 Considering the similarities between the peptide patterns of the orogastroduodenal digests of OM and dOM, 17 representative peptides, common to both hydrolysates, were selected as explained in the method section and chemically synthetized. Their IgE binding is shown in Figure 5. The highest IgE-binding (between 70 and 100% of the patients) corresponded to the peptide OM (80-89) and to the region 133-180 [OM (133-148), OM (146-161) and OM (168-180)], moderate binding (40-70% of the patients) to the regions 36-61 [OM (36-45), OM (41-53) and OM (45-61)] and 100-122 [OM (100-110) and OM (109-122)], whereas low binding (<40% patients) was attributed to the peptides OM (25-38), OM (90-103), OM (114-129) and OM (122-140). Peptides OM (19-38), OM (54-68), OM (108-118) and OM (179-186) did not react with any of the 10 patient´s sera employed in the study.

## Discussion

OM was successfully deglycosilated using microbial PNGase F (Figure 1a and 1b), an amidase that cleaves between GlcNAc and Asn residues of high mannose, hybrid, and complex oligosaccharides from *N*-linked glycoproteins, without secondary or tertiary structural changes as determined by CD spectroscopy (Figure 1c and 1d). Previous reports, had studied the effect of glycosylation on reduced and alkylated OM [5] or on individual domains of the protein [6,7]. However, because of the importance that conformational epitopes exert in some patients [5,25], the preservation of the structural stability and integrity is very important when assessing the immunoreactivity of OM.

In the present study, 80% of the patients showed lower IgE binding to dOM as compared with OM (Figure 2a). The maintenance of the secondary and tertiary structures of the full native form suggests that the differences in IgE reactivity found between the glycosylated and deglycosylated forms are not merely due to changes in protein structure induced by glycans. Furthermore, in the case of some patients, IgE reactivity to OM could not be inhibited by pre-incubation with dOM (Figure 2c-l), what indicates that these patients might be sensitized not only to the peptide epitopes, but also to carbohydrate-containing structures, although cross-reactivity between carbohydrate-containing and amino acid structures could not be excluded. Evidence for the sensitizing potential in humans of glycosylated allergens from major royal jelly proteins, beyond carbohydrate-based cross-reactivity, has been provided [26]. However, mice subcutaneously sensitized to OM third domains with and without carbohydrate do not show differences in IgE-binding towards each other, showing a high degree of cross-reactivity between the glycosylated and deglycosylated forms [27]. At this respect, it should be taken into account that a considerably variability among egg allergic patients in their IgE binding to different linear and conformational epitopes of OM has been described [5,28] that likely reflects sensitization to different OM forms.

In accordance with the lower IgE binding, we found a subtle reduction in the percentage of activated basophils after incubation with dOM as compared to OM (Figure 3). There are several examples of IgE antibodies against plant food N-glycans that exert biological activity in basophil activation assays [29], but fail to demonstrate their capacity to elicit an immune response and trigger clinical symptoms [9]. On the other hand, other authors have obtained results that support the immunological and clinical relevance of the carbohydrate determinants in allergens. A recent report from Chiang et al. [30] states the direct implication of low-molecular-weight oligosaccharides in IgE-mediated anaphylaxis to cow´s milk formula supplemented with prebiotics. 

 The simulated digestion experiments showed that OM and dOM were not affected by *in vitro* oral digestion, but completely degraded by gastric digestion (Figure 4a and 4b). A profile of 3 main degradation products with molecular masses of ~25, ~15 and <10 kDa was found in the 60 min gastric digests of OM, while dOM was digested more extensively and only bands of ≤ 15 kDa were present at that time. This might be a direct consequence of the removal of the carbohydrate chains that would allow a most efficient hydrolysis by pepsin.

Kovacs-Nolan et al. [12] described the rapid degradation of intact OM during simulated gastric digestion with the formation of large fragments that could act as allergens, albeit they exhibit reduced IgE-binding activity as compared with the native protein. Two of these, with molecular masses of ~24 and ~18 kDa were identified as OM (21-133) and OM (134-186) [12]. The bonds Leu_20_-Val_21_ and Ala_133_-Val_134_ were cleaved by pepsin in our system (Figure 5) and thus, any of these could correspond with the, likely glycosylated, IgE-binding broad ~25 kDa band of the OM digest (Figure 4c) The absence of equivalent bands in the dOM digests after 7 min of hydrolysis could account for their lower immunoreactivity, only attributable to the lower molecular mass products (Figure 4d). Takagi et al. [14] reported the formation of IgE-binding pepsin degradation products of 7 and 4.5 kDa, what reinforces the hypothesis that patients that positively react to digestion resistant fragments are unlikely to outgrow egg-allergy [31].

 Little to no change in the band pattern was observed during the duodenal phase of digestion, likely because the peptides released by pepsin action retain trypsin inhibitory activity that helps to maintain OM peptide fragment integrity [12]. Following duodenal digestion, the fragments of ~15 and ≤10 kDa that persisted in the digests of OM and dOM could be partially responsible for their residual IgE binding, which was similarly reduced, although not eliminated upon gastrointestinal digestion (Figure 4c and 4d). In any case, IgE-binding of the gastroduodenal digests was very low (<5% of that of the intact protein), what is in agreement with previous results [13]. 

 A further investigation on whether the peptides resulting from orogastrointestinal digestion of OM contained IgE-binding epitopes revealed that two regions of the protein, that is those within the residues 80-89 and 133-180, were recognized by more than 70% of the allergic patients studied, while from 40 to 70% of the patients recognized two other regions, 36-61 and 100-122 (Figure 5). Most of these high-frequency IgE-binding peptides were either totally or partially coincident with previously described epitopes [5,7,25,28,32,33]. However, there are not only numerous IgE binding epitopes distributed along the whole OM structure, but also very many differences in epitope recognition among patients depending on their sensitivity to the allergen [28]. Furthermore, the length of the peptide and the nature of the nearby amino acids also determine the affinity for IgE antibodies. It should be mentioned that longer peptides were found in the gastric and duodenal digests of OM as compared to dOM, due to the more extensive degradation of the latter. In any case, and in view of the peptides identified in the digests of OM and dOM, multiple epitopes within each domain would remain linked by disulfide bonds despite proteolytic cleavage, giving rise to complex sequences which may have the ability to cross-link several IgE molecules and activate effector cells. Nevertheless, according to Martos et al. [17], simulated gastrointestinal digestion of OM greatly diminishes its basophyl activating capacity, therefore, considering the allergenicity of OM, the possibility that digestion may promote its sensitizing potential or abrogate its tolerizating capacity should be considered.

In conclusion, this work provides evidence for an enhanced IgE reactivity towards carbohydrate containing OM in some egg-allergic patients that can be due to cross-sensitization, but also to sensitization to the glycosylated components. In addition to a direct implication of the carbohydrate chains of OM on its IgE binding, whose clinical relevance remains to be established, they contribute to an increased resistance to proteolysis, particularly during the first stages of gastric digestion, which may play a role in its allergenic potency. Although the residual IgE binding of the *in vitro* digests of OM and dOM was low, the evaluation of the presence of potential epitopes among the nonglycosylated orogastroduodenal products of digestion of OM and dOM revealed the presence of high-frequency IgE-binding fragments that could remain linked by disulphide bonds.
